# Basal Intestinal Morphology, Immunolocalization of Leptin and Ghrelin and Their Receptors in Newborn Wistar Rats after Prenatal Exposure to Fumonisins

**DOI:** 10.3390/ani13091538

**Published:** 2023-05-04

**Authors:** Ewa Tomaszewska, Halyna Rudyk, Piotr Dobrowolski, Marcin B. Arciszewski, Janine Donaldson, Katarzyna Kras, Beata Abramowicz, Damian Kuc, Siemowit Muszyński

**Affiliations:** 1 Department of Animal Physiology, Faculty of Veterinary Medicine, University of Life Sciences in Lublin, 20-950 Lublin, Poland; 2 Laboratory of Feed Additives and Premixtures Control, State Research Control Institute of Veterinary Drugs and Feed Additives, 79000 Lviv, Ukraine; 3 Department of Functional Anatomy and Cytobiology, Faculty of Biology and Biotechnology, Maria Curie-Sklodowska University, 20-033 Lublin, Poland; piotr.dobrowolski@mail.umcs.pl; 4 Department of Animal Anatomy and Histology, Faculty of Veterinary Medicine, University of Life Sciences in Lublin, 20-950 Lublin, Poland; mb.arciszewski@wp.pl (M.B.A.); katarzyna.kras@up.lublin.pl (K.K.); 5 School of Physiology, Faculty of Health Sciences, University of the Witwatersrand, Parktown, Johannesburg 2193, South Africa; janine.donaldson@wits.ac.za; 6 Department and Clinic of Animal Internal Diseases, Faculty of Veterinary Medicine, University of Life Sciences in Lublin, 20-612 Lublin, Poland; beata.abramowicz@up.lublin.pl; 7 Chair and Department of Developmental Dentistry, Medical University of Lublin, 20-081 Lublin, Poland; damian.kuc@umlub.pl; 8 Department of Biophysics, Faculty of Environmental Biology, University of Life Sciences in Lublin, 20-950 Lublin, Poland; siemowit.muszynski@up.lublin.pl

**Keywords:** fumonisins, intestine, duodenal morphology, prenatal exposure

## Abstract

**Simple Summary:**

Mycotoxin-contaminated feed is a current, worldwide problem. Mold-contaminated cereal is rich in heat-resistant and harmful metabolites such as fumonisins. The amount of fumonisins consumed as part of animal feed, including livestock feed, is unknown, and thus their influence on animal health, the costs associated with veterinary health care and the associated economic losses in breeding are impossible to calculate. Prenatal exposure of fetuses to fumonisins, through exposure of the mothers to fumonisins during pregnancy, is an important problem during breeding, often resulting in disturbed development of offspring in the postnatal period as a result. Thus, studies focused on the effects of fumonisin-exposure during the prenatal period on postnatal development are required.

**Abstract:**

Animal feed is very frequently contaminated with different types of mold, the metabolites of which are toxic to living organisms. Mold-contaminated cereal is rich in heat-resistant and harmful metabolites such as fumonisins (FBs). The amount of FBs consumed as part of animal feed, including livestock feed, is unknown. Therefore, this study aimed to evaluate the effects of maternal oral FB intoxication on basal duodenum morphology and the immunolocalization of gut hormones responsible for food intake (leptin and ghrelin), as well as their receptors, in newborn rat offspring. Pregnant Wistar rats were randomly allocated to one of three groups: a control group or one of two FB-intoxicated groups (60 or 90 mg FB/kg b.w., respectively). Basal morphological duodenal parameters changed in a dose- and sex-dependent manner. The intensity of the ghrelin immunoreaction was unchanged in females, while in males it increased after FB exposure (60 mg/kg b.w.), with a simultaneous decrease in expression of the ghrelin receptor. Leptin and its receptor immunoreaction intensity was decreased in both sexes following FB exposure. The current study highlighted the potential involvement of intestinal ghrelin and leptin in the metabolic disturbances observed later in life in offspring that were prenatally exposed to fumonisins.

## 1. Introduction

Animal feed is very frequently contaminated with different types of mold, the metabolites of which are toxic to living organisms [1]. *Fusarium* is a common genus of imperfect fungi which produces heat-resistant fumonisins [2,3]. Although *Fusarium* produces the A, B, C, and P types of fumonisins, type B (FB) is the most common environmental threat, which includes two types: FB1 and its cytotoxic analog, FB2. Both types of FB inhibit sphingosine N-acyltransferase [4], and in addition, FB2 specifically inhibits the protein serine/threonine phosphatase. [5]. They disturb sphingolipid metabolism [6], and besides their carcinogenicity, they are also nephrotoxic and cause hepatocarcinogenesis, immunosuppression and neurotoxicity [7,8,9]. Signs of FB-induced intoxication differ depending on the animal species, their age and sex, as well as on the FB dose, duration, and route of FB exposure. There are observed non-species-specific symptoms in sheep, pigs, poultry, horses, and rodents; and organ-specific symptoms in the lungs and esophagus of pigs or in the brains of horses [10,11,12]. Some animals, such as ruminants, are resistant to the effects of FB intoxication, since the rumen microbiota degrade the mycotoxins [13]. Assessment of the dietary intake of FBs is difficult since (1) their concentration in feed components is unknown, (2) infrastructure is contaminated, and (3) FBs are also produced by *Aspergillus niger*, a mold which is commonly found in soil, water and fecal matter [14]. Although the acceptable level of FBs in feedstuff is regulated by the EU Commission legislation within the European Union [15,16] and by the FDA in the USA [17], complete control of the amount of FBs in animal feed is impossible and the effects of FB exposure are still not fully known. Economic costs of veterinary care and livestock losses due to exposure to mycotoxins are impossible to calculate, because animals are sometimes already exposed to FBs prenatally and the subsequent FB-induced developmental disturbances result in many negative long-term effects later in life. Prenatal FB exposure results not only in the altered development, structure, and function of bone or the gastrointestinal tract (liver and intestines, including the enteric nervous system), impaired hematopoiesis, and skeletal muscle inflammation, it also leads to endocrine alterations, resulting in disproportional development [18,19,20,21,22,23].

Considering the indirect impact of maternal FB exposure during pregnancy on fetal development and the resulting health problems in offspring during postnatal life, it is crucial to investigate the effects of FB exposure during pregnancy not just on intestinal development but also on the gut hormones that regulate food intake in newborn offspring. It is in line with the hypothesis of the prenatal origin of health and disease according to fetal programming [24,25]. New evidence suggests that prenatal exposure to FBs could lead to the development of overweight and diabetes by altering metabolism [19], involving changes in hormonal gastrointestinal regulation of food intake.

We hypothesize that prenatal FB exposure not only affects the intestinal morphology but also can alter the immunolocalization of basal gut hormones involved in food intake, resulting in postnatal developmental impairment.

Therefore, the objective of the present study was to evaluate the impact of maternal oral FB intoxication on the small intestine of newborn rat offspring. Specifically, the study analyzed changes in the basal duodenum morphology and immunolocalization of gut hormones responsible for food intake (leptin and ghrelin) and their receptors. These analyses are essential for gaining a better understanding of the consequences of FB exposure during pregnancy on the disproportional postnatal development of rat offspring.

## 2. Materials and Methods

The animal study protocol (#132 676-Adm/08/2020) was approved by the Institutional Review Board of the State Scientific Research Control Institute of Veterinary Medicinal Products and Feed Additives (SCIVP) in Lviv, Ukraine. The experiment was performed in accordance with the EU Directive 2010/63/EU.

### 2.1. Preparation of Fumonisins Stock Solution

FBs were produced in vitro using *F. moniliforme* on a maize grain medium, as previously described [26]. Briefly, the process included inoculating autoclaved, coarsely cracked grains with *F. moniliforme* cultures and allowing them to cultivate for 4 weeks at 24 °C. The resulting contaminated maize was then autoclaved, dried, ground, and subjected to liquid chromatography to analyze FB1 and FB2 levels, which showed a ratio of FB1 to FB2 of 3:1 (73% to 27%). FBs were subsequently extracted from the ground grains using ethanol solution, quantified with an ELISA kit (R-Biopharm, Darmstadt, Germany), and concentrated to a 100 mg/mL FBs extract stock solution. During the experiment, to achieve the required concentrations in 0.5 mL, the FB extract stock was diluted in a 0.9% saline solution based on daily measurements of the body weight of individual pregnant dams.

### 2.2. Animals and Experimental Design

Pregnant Wistar rats, at the age of five weeks (*n* = 18), were housed individually in polypropylene cages (380 × 200 × 590 mm). The rats were kept at a temperature of 21 ± 3 °C and a humidity of 55 ± 5% under a 12 h light/dark cycle. A standard diet for laboratory rat was provided ad libitum, with unlimited access to water. After an acclimatization period, the pregnant dams were randomly assigned to one of three groups (with six rats in each group): a control group (the FB 0 group) or one of two FB-intoxicated groups, which received FBs at a dose of either 60 mg FB/kg b.w. (the FB 60 group) or 90 mg FB/kg b.w. (the FB 90 group), respectively. FBs at a dose of 90 mg FB/kg b.w., which is equivalent to 1/10 of the established LD50 value, is sufficient to cause sub-clinical intoxication in adolescent rats, while the 60 mg FB/kg b.w. dose, which is equivalent to 1/15 of the established LD50 dose, does not elicit clinical or subclinical signs in adolescent rats [18,20,27]; however, both doses resulted in higher body weight at weaning with probable insulin resistance noted mainly following the 60 mg/kg b.w. dose [19]. The two doses are routinely used in developmental studies (omitting the first 6 days of pregnancy) carried out at SCIVP, Lviv, Ukraine [27,28].

The FB mixture, diluted from FB extract stock in 0.5 mL of 0.9% saline, was given to the dams daily from the 7th day of pregnancy to parturition, intragastrically via oral gavage. Pregnant control animals were administered saline solution at the same volume. All pregnant females were under veterinary care. After natural parturition, all newborn offspring were allocated to the following groups, in line with their mothers: the FB 0, FB 60 or FB 90, and two newborn offspring from each mother (one male and one female, *n* = 6 males and *n* = 6 females in total) were weighed and euthanized (CO_2_ inhalation). Immediately after euthanasia, the duodenum was carefully dissected out and fixed in 10% buffered neutral formalin solution for morphometrical assessment and immunohistochemical evaluations.

### 2.3. Tissue Sampling and Morphometrical Analysis

**Figure 1:**
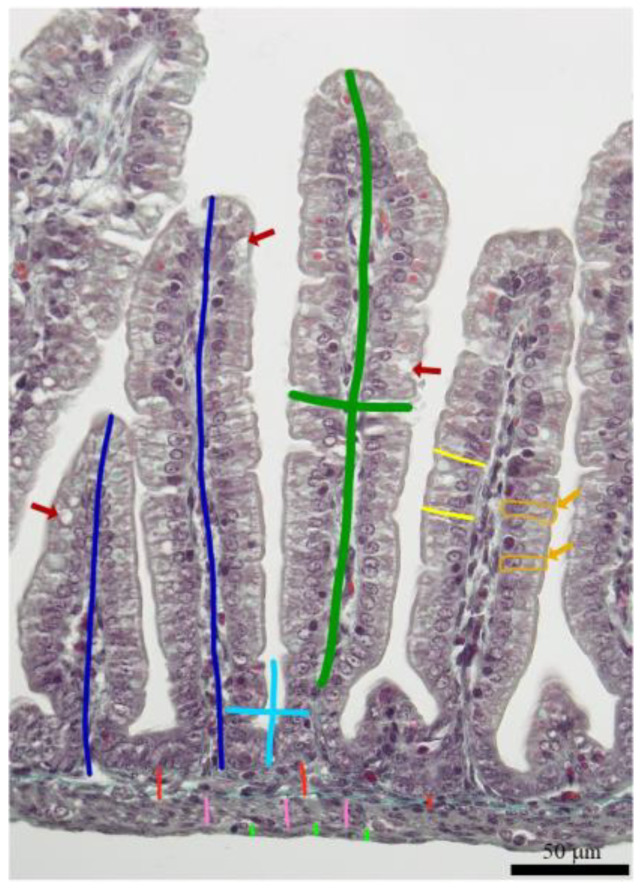
Representative image of duodenal wall showing measurement scheme of morphological parameters: the length and thickness of intestinal villus (green line), the depth and thickness of the crypt (light blue line), thickness of the villus epithelium (light yellow line), mucosa (dark blue line), submucosa (red line), and longitudinal (light green) and circular (pink line) muscle layers. Orange arrows show enterocytes (additionally outlined by orange line), while red arrows show goblet cells.

After being fixed in 10% formalin for 24 h, intestine samples were washed in water, dehydrated in a series of graded ethanol solutions (30, 50, 70, 80, 90, 96, and 99.8%). They were then paraffin-embedded, cut into 5 µm sections, mounted on a microscope slide and stained with Goldner’s trichrome staining, a histological staining technique used in pathology to visualize and differentiate various tissue components. It typically stains collagen fibers green, muscle fibers red, and nuclei black or brown, allowing for the identification and assessment of connective tissue, muscle tissue, and cell nuclei in histological sections [29,30]. The specimens were observed under standard bright illumination with an optical microscope (BX63 and CX43, Olympus, Tokyo, Japan) using the 20× objective. The length and thickness of intestinal villi, as well as the length and thickness of the crypts, thickness of the villus epithelium, mucosa, submucosa, and both myenterons (longitudinal and circular muscle layer), and the number of villi and crypts per mm of mucosa, were measured as described in detail previously [21,28] (Figure 1). The villus height/crypt depth ratio was calculated for individual villi–crypt pairs. The number of enterocytes and goblet cells per 100 µm of villus was also determined [31,32]. Special attention was paid to the correct orientation, and only sections containing longitudinally cut villi and crypts were included in the analyses. For each parameter, 10 replicate measurements were made per animal. The analyses were performed using ImageJ software (version 1.51k) [33].

### 2.4. Leptin, Ghrelin and Their Receptors Immunostaining

The immunohistochemical (IHC) stain was performed with rabbit anti-leptin, anti-leptin receptor, anti-ghrelin, and anti-ghrelin receptor as primary antibodies (#DF8583, #DF7139, #DF6389, #DF2794, respectively, all Affinity Biosciences, Jiangsu, China), diluted (1:300) in Diamond antibody diluent (Cell Marque Corp., Rocklin, CA, USA). The sections were then processed with a ready-to-use, two-step detection system of poly-HRP-anti-mouse/rabbit IgG (#IM-DPVB110HRP, BrightVision, Immunologic WellMed B.V., Duiven, Netherlands), developed in DAB (#D5905, Sigma-Aldrich, St. Louis, MO, USA) and counterstained with Mayer’s hematoxylin (Patho, Mar-Four, Konstantynów Łódzki, Poland) [34]. The IHC images, acquired using a bright-field microscope (BX-61, Olympus, Tokyo, Japan) and the 20× objective (200× total magnification), were analyzed using an ImageJ-compatible plugin for analyzing cytoplasmic staining patterns by assigning a histogram profiling and scoring the deconvoluted DAB images using IHC Profiler [35], an open-source plugin for semiquantitative and quantitative evaluation and automated scoring of immunohistochemistry images. An optical density (OD) quantitative score was calculated according to algebraic formula previously described by Jafari and Hunger [36].The measurements were carried out separately for villi and crypts in five randomly selected areas of the positive signal by an associate who was blinded to the treatment.

### 2.5. Statistical Analysis

The data collected in the study were analyzed using the statistical software system STATISTICA (version 13, StatSoft, Inc., Tulsa, OK, USA) and GraphPad Prism (version 9.5.1.733, GraphPad Software, San Diego, CA, USA). An experimental unit consisted of a single rat, so measurements were averaged per animal before statistical analysis. The data for males and females were analyzed separately, and all data were confirmed to be normally distributed through the Shapiro–Wilk normality test. The analysis was conducted using a one-way analysis of variance (ANOVA) based on the model: Y_ij_ = m + D_i_ + e_ij_, where Y represents a single observation, m represents the general mean, D represents the effect of the FB exposure (i.e., 0, 60, and 90 mg FB/kg b.w.), and e represents the error. Post hoc comparisons were conducted using the Tukey HSD test. Additionally, to determine the linear and quadratic effects among the means, an orthogonal contrast analysis was conducted.

## 3. Results

### 3.1. Duodenal Basal Morphology

Basal morphological changes after prenatal/maternal fumonisin exposure in female newborn rat are presented in Table 1. Maternal FB exposure, irrespective of the dose, resulted in a decrease in the thickness of the longitudinal lamina, compared to that observed in the control group, with both linear and quadratic effects being statistically significant (linear and quadratic, *p* < 0.001 and *p* = 0.002, respectively). The thicknesses of circular muscle layer, villus and villus epithelium decreased after prenatal FB exposure, irrespective of FB dose, compared to the control group (linear, *p* = 0.008, *p* = 0.001, and *p* < 0.001, respectively). Total crypt number was significantly lower in newborn offspring exposed to fumonisins at a dose of 60 mg/kg b.w., compared to that of the other groups (linear, *p* < 0.001). No other changes in serum parameters were observed.

In the case of male newborn offspring, prenatal exposure to FBs resulted in a trend of decreased thickness of the longitudinal muscular lamina (*p* < 0.001). Moreover, crypt depth (*p* = 0.025), goblet cell number per 100 µm of villus (*p* < 0.001), villi length/crypt depth ratio, and villus epithelium thickness (*p* < 0.001) decreased linearly, while mucosa thickness, crypt thickness, and villus length decreased linearly and quadratically (*p* = 0.028 and *p* = 0.024; *p* < 0.001 and *p* = 0.008, respectively) following FB exposure (Table 2).

### 3.2. Ghrelin, Leptin, and Their Receptors

**Figure 2:**
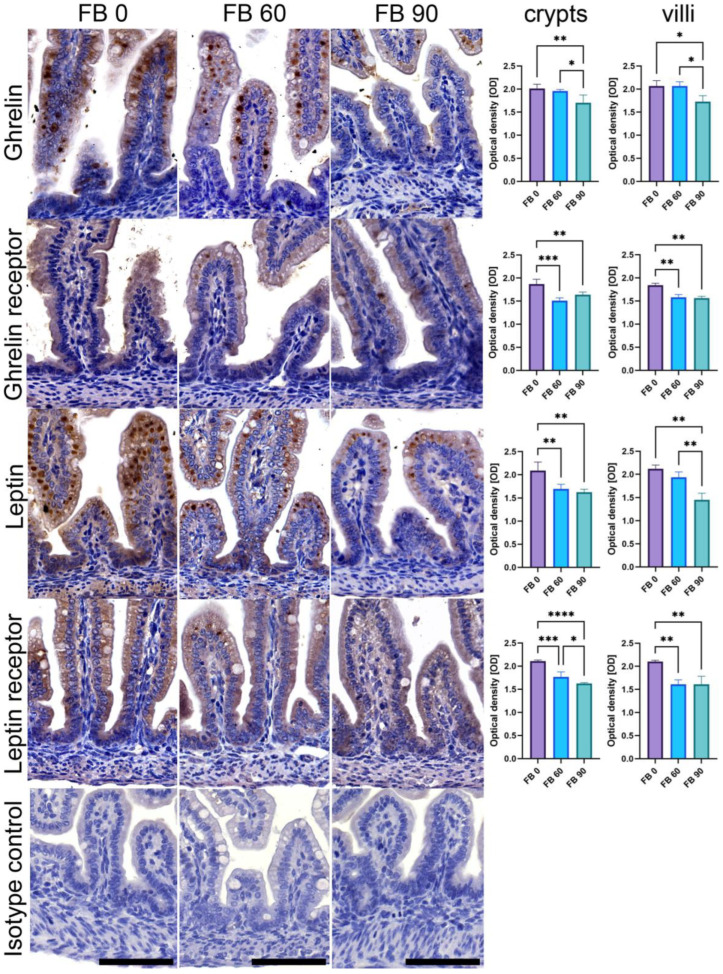
Representative images and quantitative analysis of the intensity of ghrelin, ghrelin receptor, leptin, and leptin receptor immunoreaction (IR) in crypts and villi of the duodenum of female newborn rat offspring after maternal exposure to fumonisins (FBs) at the doses of 0, 60, or 90 mg/kg b.w. Bottom isotype row shows IHC IR control incubated without a primary antibody. All of the images were taken at the same magnification; scale bars represent 50 μm. Bar charts: intensity of immunoreaction (IR) to specific antibody was measured in crypts and villi using optical density (OD) measurements in the microscopic images. Results are presented as mean and SD. Statistically significant differences between groups are indicated by * *p*-value < 0.05; ** *p*-value < 0.01; *** *p*-value < 0.001; **** *p*-value < 0.0001.

As shown in Figure 2, the optical density (OD) of the ghrelin immunoreactivity (IR) in crypts and villi of female newborn rat offspring significantly decreased following prenatal exposure to 90 mg/kg b.w. FB (linear contrast in crypts and villi, *p* < 0.001 for both), with a greater decrease in crypts when compared to the control group. The OD of the ghrelin receptor IR in crypts showed a significant decrease regardless of the FB dose, with a linear and quadratic trend in crypts (*p* < 0.001 for both) and a linear trend in villi (*p* < 0.001). The OD of the leptin IR decreased following FB exposure in crypts (linearly and quadratically, *p* < 0.001 for both), while in villi the OD of the leptin IR decreased linearly (*p* < 0.001), showing a significant difference only for the FB 90 group when compared to the control. The OD of the leptin receptor IR decreased linearly in crypts (*p* < 0.001) with the lowest value observed in the FB 90 group, while in the villi the decrease was linear and quadratic (*p* < 0.001 for both), but without a difference between the FB-intoxicated groups.

**Figure 3:**
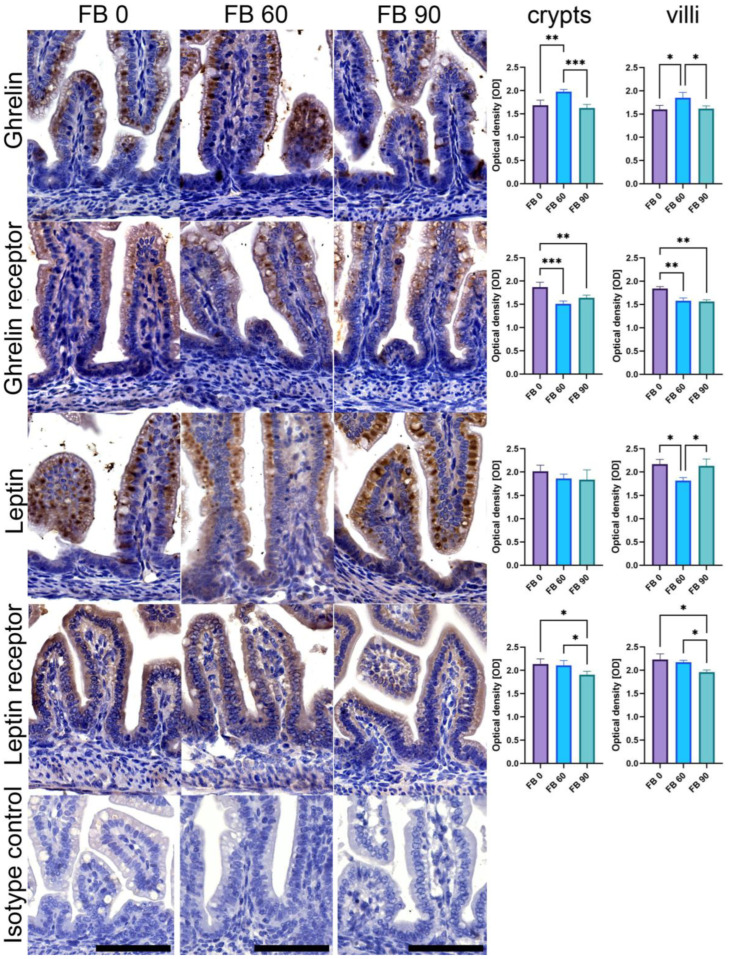
Representative images and quantitative analysis of the intensity of ghrelin, ghrelin receptor, leptin, and leptin receptor immunoreaction (IR) in crypts and villi of the duodenum of female newborn rat offspring after maternal exposure to fumonisins (FBs) at the doses of 0, 60, or 90 mg/kg b.w. Bottom isotype row shows IHC IR control incubated without a primary antibody. All of the images were taken at the same magnification; scale bars represent 50 μm. Bar charts: intensity of immunoreaction (IR) to specific antibody was measured in crypts and villi using optical density (OD) measurements in the microscopic images. Results are presented as mean and SD. Statistically significant differences between groups are indicated by * *p*-value < 0.05; ** *p*-value < 0.01; *** *p*-value < 0.001.

Figure 3 shows representative images and results of quantitative analysis of the OD of IR observed in male offspring. The OD of the ghrelin IR in crypts showed a quadratic trend (*p* < 0.001), with the highest OD observed in the FB 60group. The OD of the ghrelin IR in villi showed the highest value in the FB 60 group as well, but both a linear (*p* = 0.033) and quadratic trend (*p* < 0.001) were observed. The OD of the ghrelin receptor IR in crypts and villi significantly decreased following prenatal exposure regardless of the FB dose. The decrease showed a linear trend (*p* < 0.001, for both) and a quadratic trend (*p* < 0.001 and *p* < 0.05 for crypts and villi, respectively). In the crypts of the duodenum of newborn male rat offspring, no effect of prenatal exposure to FBs was observed on the OD of the leptin IR. In the villi, a quadratic trend was noted (*p* < 0.001), with the lowest OD observed in the FB 60 group. The OD of the leptin receptor IR significantly decreased in the crypts and villi of the FB 90 group when compared to that observed in the FB 0 and FB 60 groups (*p* < 0.05).

## 4. Discussion

Fumonisins have been shown to inhibit lipid biosynthesis-related proteins (ceramide synthase is inhibited by all fumonisins, whereas the serine/threonine phosphatase is only inhibited by FB2), resulting in disturbances in sphingolipid synthesis, the biological effects of which include altered cellular differentiation, cell proliferation, programmed cell death, apoptosis, and inflammation [18,19,27]. Maternal nutrition has been shown to be a very strong determinant of the prenatal formation of all fetal structures, as well as their function, which in turn influences all aspects of postnatal life [24,25]. Postnatal functioning of living organisms is dependent not only on the postnatal development of all the structures and systems of the organism (skeletal system determining movement or gastrointestinal system determining the whole metabolism) which in turn influences physical activity, but also on their mental health, which determines their position in the environment and in their peer and social groups. Thus, prenatal nutrition could be a factor that will determine which traits will be preserved and passed on epigenetically to the next generation. Many harmful or toxic substances, including fumonisins, are often present in the diet of animals, including that of pregnant animals [12,37]. To address this concern, the European Commission has released recommendations on the acceptable levels of fumonisins in maize and animal feed [16]. Prenatal development is characterized by a period of developmental plasticity during which the formation and maturation of many internal organs and systems occurs, which are crucial for postnatal health [25,38,39]. Fumonisins cross the placental barrier and exert many negative effects, dependent on the dose, duration of exposure, and time at which the fetuses are exposed [40,41]. During the current study, fumonisins were administered from the 6th day of the rats’ pregnancy, when the small intestine, including the duodenum, is not yet formed [42]. Smooth muscle and epithelial cells of the small intestine are not yet differentiated on day 15.5; however, the intestinal tight junctions are formed. Circular and longitudinal muscular layers develop on day 16.5, and on day 17.5 the first duodenal villi appear, epithelial cells differentiate to enterocytes, and the first endocrine cells develop. The villi epithelium is complete on day 18.5, and at the same time goblet cells appear. The last 3 days before birth are crucial for completion of intestinal development, and the intestinal mucosa becomes comparable to that found postnatally, with a significant increase in intestinal villi and microvilli in the brush border. On day 21, villi shape and size are comparable to that characteristic of postnatal structures [42]. Newborn rats have a short intestine (20 cm), weighing about 0.066 g, and during the first 21 days of postnatal life there is a large increase in intestinal weight (about eightfold) and a twofold increase in length [43]. The duodenum is the shortest and most proximal segment of the small intestine and the initial site of contact of food with the gastric secretions, bile and digestive enzymes. The duodenum plays an important role in the alkalinization of gastric chyme, which is important in the prevention of mucosal damage, because duodenal luminal pH fluctuates between 2 and 7 due to the presence of gastric acid. The acid is neutralized not only by bicarbonate, but also by the mucus produced by the goblet cells. The duodenum is also involved in the control and regulation of digestion, absorption, and motility, since it produces hormones which are critical for the coordination of all these processes [44].

The current study was designed to take place during the period of development when the most important alterations occur in the intestine, and the results showed that prenatal fumonisins exposure reduced mucosa thickness in a sex-dependent manner. This FB-induced change could not only influence secretion, digestion, and absorption, but also the protective functions of the intestine, involving non-specific (defense against injury) and specific immunity (involving mucosa-associated lymphoid tissue) [45]. The negative effects of prenatal fumonisin exposure are still not fully known and there are very few previous studies which present such effects, making it difficult for us to discuss them.

The only available study on rats prenatally exposed to fumonisins at the same doses as those used in the present study (60 and 90 mg/kg b.w./day) found that the reduced mucosal thickness observed in the duodenum of offspring at birth (such as that observed in the rats in the current study) seems to be temporary, since it disappears at weaning [21]. However, this study did not differentiate between males and females upon weaning. Another previous study observed no effect of FB exposure on mucosal thickness; however, it was performed on adolescent male rats [28].

The smooth muscle fibers of the muscular lamina are arranged in two different layers (the inner circular and outer longitudinal) and determine the spiral passage of chyme to the periphery. Physiologically, the muscular lamina is the thickest in the proximal small intestine and it thins as it moves away from the pylorus. The present study only focused on the duodenum, where a thinning of both muscular layers was noted. Although we are not sure how prenatal FB exposure affects the other segments of the small intestine in rats, we can assume that it could lead to disturbances in the mixing of the chyme with digestive juices and in the movement of the chyme forwards, ultimately leading to incomplete emptying of the intestine. Moreover, it is unknown how prenatal exposure to fumonisins and the consequent thinning of the muscular lamina noted in female newborn rats could influence the basic electrical rhythm (BER), triggered in the interstitial pacemaker cells of Cajal, as well as the other physiological properties of the duodenum, such as excitability and contractility, in which the muscular layer plays a significant role [46].

Crypts play a role in the renewal and regeneration of the villi epithelium, because immature enterocytes undergo proliferation and then rise from crypts and move along the villi and further undergo exfoliation on the villus’s tip. When proliferation and exfoliation are in equilibrium, proper villi length and shape are maintained [47]. Changes in villi morphology at weaning, when shortening is observed, have been shown to be temporary and are associated with the change in feeding from liquids to solids [48].

The current study showed that prenatal fumonisin exposure resulted in a reduction in the length and thickness of the villi. These results are in agreement with a previous study with a similar study design, where narrower villi were observed in weaned rats following FB exposure [21]. Similar effects were observed in previous studies, with shorter villi observed in adolescent male rats [28] and villi atrophy in laying hens [26] following FB exposure. Other studies also confirmed the negative effects of fumonisins on intestinal villi, irrespective of the species or fumonisin dose [49,50,51,52,53]. Additionally, a linear decrease in villi/crypt ratio with an increase in fumonisin dose could indicate villi atrophy [54].

The current study not only showed a reduction in thickness and narrowing of the newborn rat villi, but also narrower and more shallow crypts. Taken together, our results are in agreement with previous studies [26,28] and indicate that fumonisins negatively impact cell proliferation and possibly intensify cell death, leading to extensive cell exfoliation on the villi tips and shortening [53,55].

Crypts are important for non-specific immunity since Paneth cells are located inside them, which produce many different bacteriocidial secretions, as well as specific endocrine amine precursor uptake and decarboxylation cells, which produce various biogenic amines or hormones which act in a paracrine or endocrine manner [56]. The secretory role of the crypts could be disturbed by prenatal fumonisin exposure, since a decrease in the number of crypts was observed in the newborn rat offspring following FB exposure. The same effect was also previously noted in weaned rats following FB exposure [21]. Taking into account the results observed in weaned rats from a previous study and the results of the current study, it seems that the reduction of the crypt number is permanent. However, this effect was not observed in adolescent rats directly intoxicated with fumonisins [28].

A decrease in the number of goblet cells and villi epithelium thickness was also observed in the current study. The villi epithelium, together with the microbiota, immune cells, goblet cells, and tight junctions, constitute the intestinal barrier [57]. Although a reduction in villi epithelium thickness has not been observed in previous studies, a reduction in the number of goblet cells has been noted and is in agreement with our results [28,51,52].

The proper development of intestinal structures is necessary for further general growth and development of organisms, and any changes in normal intestinal development can be associated with disease and impaired growth later in life [58]. As previously reported, newborn offspring from dams exposed to fumonisins, at a dose of 60 or 90 mg/kg b.w., weighed significantly less compared to control offspring, and the effects were sex-dependent. Prenatal FB exposure reduced term body weight in males, irrespective of fumonisin dose, while in females this effect was only observed after exposure to the higher FB dose (90 mg/kg b.w.) [18]. This fumonisin-induced effect on body weight and the weight of all vital organs was still observed at weaning in a sex-dependent manner [19]. Additionally, the disproportionate neonatal development observed in offspring at weaning, following maternal fumonisin exposure, has been linked with hormonal dysregulation, including that of growth hormone and insulin. Taking into account the disproportional growth, final body weight, glucose, and insulin concentration, the risk of diabetes mellitus and obesity should be considered [19].

Gut hormones, leptin and ghrelin, play a role in the regulation of feed intake. Ghrelin is an orexigenic hormone, synthesized in the stomach and other segments of the small intestine, and released directly into the bloodstream, although during the fetal period it is also expressed in the pancreas [59,60]. Ghrelin has been shown to stimulate appetite and worsen glucose tolerance in rats through a reduction in insulin sensitivity, leading to an increase in plasma glucose [61]. Food intake rapidly decreases plasma ghrelin concentrations and a decrease in ghrelin concentrations are accompanied by a simultaneous increase in leptin concentrations during obesity. However, lowered plasma ghrelin concentrations are sufficient to maintain ghrelin’s blood glucose-enhancing effect and it can even be paradoxically enhanced in obesity. This effect is considered a physiological adaptation to this state involving these two hormones [62]. Leptin, which is also produced in the stomach, has various biological effects [63]. It not only initiates puberty, participates in inflammatory and immune responses, angiogenesis, hematopoiesis and bone formation, but also plays a regulatory role in energy homeostasis. Leptin is important in the control of body weight through the stimulation of metabolic rate and the suppression of food intake, which has also been demonstrated in rodents [64].

Since ghrelin and leptin play a regulatory role in food intake through the gut–brain axis [65], it is important to know what effects prenatal fumonisin exposure has on the immunolocalization of leptin and ghrelin and their receptors in the duodenum in newborn offspring, which was previously unknown.

The current study showed that the OD of the ghrelin IR was unchanged in females and increased in males following FB exposure at a dose of 60 mg/kg b.w. with a simultaneous decrease in the ghrelin receptor. On the other hand, the OD of the leptin IR and that of its receptor, decreased in both sexes. Ideally, the mRNA gene expression and Western blot analyses of protein levels should also be performed, as well as the measurement of the concentration of these hormones in peripheral blood, to comprehensively examine all potential mechanisms of prenatal FB exposure and its effects on postnatal animal development [19], which could be in line with the Developmental Origin of Health and Disease theory which suggests that disturbances in the intrauterine environment can increase the risk of developing various diseases (such as diabetes and obesity) later in life [66].

To support these findings, further studies should be conducted to investigate whether prenatal exposure to fumonisins can trigger obesity and diabetes. Given the size of the newborn rats and the amount of samples we were able to collect, future studies should likely involve larger animals.

## 5. Conclusions

The present study is the first to suggest that disturbances in the levels of intestinal ghrelin and leptin and their receptors could contribute to the development of metabolic abnormalities in offspring, at a later stage of life, as a result of prenatal exposure to fumonisins. Due to the common presence of fumonisin in feed and the widespread risk of prenatal intoxication, research should be continued, including studies of other hormonal regulators of food intake.

## Figures and Tables

**Table 1:** The basal morphology of the duodenum of female newborn rat offspring after maternal exposure to fumonisins (FBs) at the doses of 0, 60, or 90 mg/kg b.w.

Dependent Variable	FB (mg/kg b.w.)			SEM	*p*-Value	*p*-Level	
	0	60	90			Linear	Quadratic
Longitudinal m. lamina thickness, μm	10.27 ^b^	5.72 ^a^	7.07 ^a^	0.653	<0.001	<0.001	0.002
Circular m. lamina thickness, μm	20.06 ^b^	12.68 ^a^	12.95 ^a^	1.823	0.018	0.008	0.107
Submucosa thickness, μm	12.63	10.89	9.54	1.199	0.223	0.090	0.898
Mucosa thickness, μm	246.78	210.66	235.79	24.661	0.572	0.622	0.320
Crypt depth, μm	37.1	34.87	39.41	3.350	0.640	0.751	0.422
Crypt thickness, µm	38.69	38.01	33.61	2.385	0.292	0.195	0.532
Villus length, µm	250.94	175.33	196.38	22.122	0.074	0.058	0.094
Villus thickness, µm	84.63 ^b^	60.52 ^a^	56.98 ^a^	5.264	0.004	0.001	0.131
Total number of villi, /mm	11.21	13.02	11.79	0.788	0.283	0.431	0.136
Total crypt number, /mm	13.73 ^b^	11.44 ^a^	14.45 ^b^	0.407	<0.001	0.826	<0.001
Villus length/crypt depth ratio	6.29	5.58	5.13	0.986	0.778	0.415	0.917
Enterocyte, per 100 µm of villus	17.04	17.06	15.82	0.622	0.298	0.244	0.419
Goblet cells, per 100 µm of villus	3.923	3.752	3.635	0.344	0.840	0.563	0.950
Villus epithelium thickness, µm	17.16 ^b^	13.52 ^a^	12.40 ^a^	0.824	0.003	<0.001	0.231

Results are presented as means. FB —fumonisin; SEM—standard error of the means. Groups with statistically significant differences (*p*-value < 0.05) are denoted with ^a^ and ^b^.

**Table 2:** The basal morphology of the duodenum of male newborn rat offspring after maternal exposure to fumonisins (FBs) at the doses of 0, 60, or 90 mg/kg b.w.

Dependent Variable	FB (mg/kg b.w.)			SEM	*p*-Value	*p*-Level	
	0	60	90			Linear	Quadratic
Longitudinal m. lamina thickness, μm	12.15	9.87	8.26	1.319	<0.001	0.054	0.837
Circular m. lamina thickness, μm	14.95	16.50	13.72	1.500	0.441	0.722	0.256
Submucosa thickness, μm	12.03	9.70	12.33	1.011	0.166	0.866	0.063
Mucosa thickness, μm	338.85 ^b^	216.27 ^a^	190.09 ^a^	13.236	<0.001	<0.001	0.009
Crypt depth, μm	37.16 ^b^	28.34 ^a^	29.65 ^a^	2.409	0.043	0.025	0.106
Crypt thickness, µm	40.75 ^b^	30.28 ^a^	34.13 ^ab^	2.336	0.020	0.028	0.024
Villus length, µm	301.69 ^b^	187.94 ^a^	160.44 ^a^	11.507	<0.001	<0.001	0.008
Villus thickness, µm	66.25	59.20	57.90	2.930	0.129	0.051	0.436
Total number of villi, /mm	11.80	9.92	10.74	0.707	0.202	0.200	0.140
Total crypt number, /mm	12.86	11.34	11.47	0.899	0.436	0.243	0.463
Villus length/crypt depth ratio	8.55 ^b^	6.92 ^a^	5.58 ^a^	0.424	<0.001	<0.001	0.784
Enterocyte, per 100 µm of villus	16.99	16.55	17.39	0.658	0.671	0.272	0.797
Goblet cells, per 100 µm of villus	6.08 ^b^	3.50 ^a^	2.86 ^a^	0.418	<0.001	<0.001	0.077
Villus epithelium thickness, µm	16.25 ^b^	13.12 ^a^	12.23 ^a^	0.561	<0.001	<0.001	0.125

Results are presented as means. FB—fumonisin; SEM—standard error of the means. Groups with statistically significant differences (*p*-value < 0.05) are denoted with ^a^ and ^b^.

## Data Availability

The data presented in this study are available on request from the corresponding authors.
